# Parkinson's disease: Is it a consequence of human brain evolution?

**DOI:** 10.1002/mds.27628

**Published:** 2019-02-13

**Authors:** Nico J. Diederich, D. James Surmeier, Toshiki Uchihara, Sten Grillner, Christopher G. Goetz

**Affiliations:** ^1^ Department of Neurosciences Centre Hospitalier de Luxembourg Luxembourg City Luxembourg; ^2^ Department of Physiology, Feinberg School of Medicine Northwestern University Chicago Illinois USA; ^3^ Neurology Clinic with Neuromorphomics Laboratory Nitobe Memorial Nakano General Hospital Tokyo Japan; ^4^ Department of Neurology and Neurological Science Tokyo Medical and Dental University Tokyo Japan; ^5^ Structural Neuropathology Tokyo Metropolitan Institute of Medical Science Tokyo Japan; ^6^ Department of Neuroscience Karolinska Institut Stockholm Sweden; ^7^ Department of Neurological Sciences Rush University, Chicago Illinois USA

## PD Is an Exclusively Human Disease

Although experimental lesions to the dopaminergic system lead to Parkinson's disease (PD)‐like motor symptoms in vertebrates extending from lamprey to primates,^1^ parkinsonism does not occur naturally in any species other than man. Aged nonhuman primates may show impaired fine motor control and reduced home cage activity, but these deficits are not sensitive to levodopa administration and are not accompanied by Lewy body (LB) burden.^2^ But why should PD be an exclusively human disease? One possibility is that the dramatic expansion of the telencephalon, particularly the neocortex, in humans creates a significant burden on subcortical circuits with which the telencephalon interacts, leading to increased vulnerability to aging, genetic mutations associated with PD, and environmental toxins.

## Humans and Telencephalization

**Figure 1:**
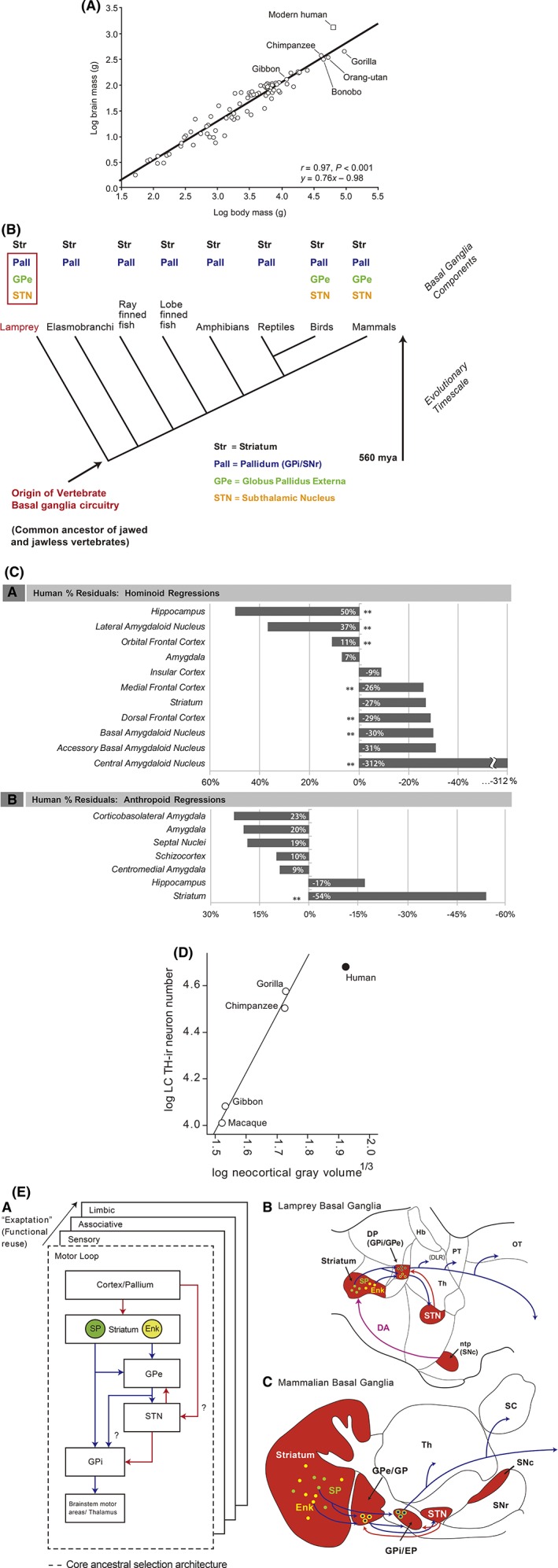
Evolutionary aspects of BG components in humans. (A) Human brain mass larger by scaling plot based on primates^40^ [with permission]. (B) Clade of the BG: all parts already present in lampreys^41^ [with permission]. (C) Negative deviations from regression line for striatum and amygdaloid nuclei in humans^17^ [with permission]. (D) Human LC (vertical axis) shows fewer neurons than expected by comparison to neocortex volume (horizontal axis); TH = tyrosine hydroxylase^19^ [with permission]. (E) Diagram showing the evolutionarily conserved functional module of the motor loop and subsequent “copy paste” of this module for other functions by the exaptation principle. Blue = GABAergic projections; red = glutamatergic projections; DP = dorsal pallidum; Enk = enkephalin; EP = entopeduncular nucleus; GP = globus pallidus; Gpe = external segment of the globus pallidus; Hb = habenula; ntp = nucleus tuberculi posterior; SP = substance P; Th = thalamus^41^ [with permission].

The human brain is approximately 3 times larger than that expected from a plot of brain weight against body size for nonhuman primates (Fig. 1A).^3^ This expansion concerns largely telencephalic structures.^3^ Expansion of the prefrontal cortex has been examined in detail, but there is ongoing debate on mechanistic aspects.^4^, ^5^ In comparison to other primates, molecular and cellular reorganization of neural circuitries in humans may be crucial.^6^ The relative growth of the human cerebral cortex may have been attributed to relaxed genetic control and the shift in the human diet from exclusively plants to a mixture of plants and nutritionally dense animal tissue, which allowed the metabolic demands of the cerebral cortex to be met without expanding the digestive tract.^7^, ^8^

Telencephalization has had obvious advantages for humans. Triggering neurodegeneration has been proposed as a downside^9^ to this growth. Could this telencephalic expansion be particularly detrimental for subcortical structures that interact with the cortex but have not grown commensurately? The basal ganglia (BG) blueprint arose nearly 500 million years ago and has been preserved throughout vertebrate phylogeny (Fig. 1B).^10^ This network helps make goal‐directed behavior and habits rapid and “automatic.” When the BG is impaired, control becomes slower and less efficient; moreover, the ability to generate rapid, stimulus‐driven, habitual motor sequences is largely lost.^11^ The largest of the BG nuclei is the striatum; it integrates information from other telencephalic structures—principally the cerebral cortex—about motor plans, internal motivational and affective states, and the external environment. Striatal activity regulates the BG interface nuclei, which then modulate other brain regions controlling movement and thought.^12^ The SNc is a key part of the BG circuit. The widely arborizing axons of these neurons innervate all parts of the BG, but have a particularly dense innervation of the striatum.^13^ Striatal processing of cortical signals depends upon this dopaminergic innervation, because it provides critical information about the outcome of actions and ongoing movement.^14^

Although ontogenetically the striatum is part of the telencephalon, in the course of human evolution, neocortical growth has been 5 times that of striatum.^15^ In fact, human striatal volume is significantly below predicted values from anthropoids (Fig. 1C). One way in which the striatum may have dealt with this “involution” is by “exaptation,” where ancestral circuits take on new jobs (Fig. 1E). Another part of the BG that has not kept pace with the cerebral cortex is the SNc. In humans, the number of SNc dopaminergic neurons per unit striatal volume is roughly one tenth that in a rodent.^16^ This means an individual SNc dopaminergic neuron innervates a much greater volume in humans. In humans, it is estimated that a single SNc axon may form 1 to 2 million synapses in the striatum—an order of magnitude greater than the number in a rodent.^16^ This extraordinary growth may be crucial to pathogenesis, given that the axon is widely thought to be the most vulnerable part of the human SNc dopaminergic neuron, beginning to degenerate in the earliest stages of PD.

Other vulnerable parts of the brain also appear to have been “left behind.” This concerns the evolutionarily oldest parts of the amygdala such as the basal, accessory basal, and central amygdaloid nuclei; the human central amygdaloid nucleus is estimated to be one third the size expected of a hominoid.^17^ In contrast, the evolutionarily younger lateral amygdaloid nucleus is almost 40% larger than expected. Of note: The cells of the older amygdalar nuclei are densely branched spiny neurons similar to the striatum, whereas the younger amygdalar nuclei contain pyramidal‐like neurons similar to the cerebral cortex.^18^ When compared to the cerebral cortex, the total number of locus coeruleus (LC) neurons is substantially lower than expected in humans (Fig. 1D).^19^ The olfactory bulb is approximately 30% as large as it should be for a primate brain.^20^ Many of the other neurons at‐risk in PD—basal forebrain cholinergic neurons, pedunculopontine cholinergic neurons, intralaminar thalamic glutamatergic neurons, raphe serotonergic neurons, and lateral hypothalamic orexin neurons—also have long, highly branched axons that innervate the cerebral cortex or regions that have been affected by the relative growth of the telencephalon.^21^

## Mitochondrial Determinants of Vulnerability

How might a highly branched, unmyelinated axon with millions of transmitter release sites increase a neuron's risk in PD? Most of the thinking around this point has focused on the bioenergetic demands associated with sustaining the electrochemical gradients necessary for spike propagation and the machinery necessary for neurotransmission. The proposition that mitochondrial oxidant stress is a driver of pathogenesis in PD is consistent with three other key pieces of evidence. First, genetic mutations that increase mitochondrial oxidant stress or impair mitochondrial quality control lead to early‐onset forms of PD. Second, environmental toxins that impair mitochondrial function increase the risk of PD. Third, mitochondrial function declines with age—the biggest risk factor for PD.^22^ However, there are counterexamples where other neurons with extensive axonal arbors, such as the neurons of the internal globus pallidus (GPi) and the cholinergic medium spiny interneurons—ones as large as those of SNc dopaminergic neurons—that do not succumb in PD.^23^Are there other distinguishing features of at‐risk neurons that might advance the “tipping point”? The best‐studied example is the SNc dopaminergic neuron. At‐risk ventral tier SNc dopaminergic neurons are slow, autonomous pacemakers with broad action potentials and large oscillations in cytosolic Ca^2+^ concentration.^24^ Although Ca^2+^ oscillations contribute to pacemaking per se, their primary function appears to be bioenergetic. With each cycle of pacemaking, plasma membrane Cav1 (L‐type) Ca^2+^ channels open and trigger the release of Ca^2+^ from intracellular stores. This release loads juxtaposed mitochondria with Ca^2+^, driving oxidative phosphorylation and adenosine triphosphate (ATP) production. This “anticipatory” or feed‐forward bioenergetic control mechanism appears to be a phylogenetically old mechanism that diminishes the chances that bouts of unpredictable, sustained activity result in ATP depletion that shuts a neuron down.^12^ For SNc dopaminergic neurons, terminal or somatodendritic ATP depletion and the failure to sustain dopamine release during periods of fight or flight would be dangerous, even disasterous, given that it would slow movement at precisely the time when survival is threatened. Why is the direct dopaminergic innervation of the cerebral cortex by the ventral tegmental area (VTA) not affected by the same process? The answer is likely to be many‐faceted given that VTA dopamine (DA) neurons have a quite different phenotype; for example, their axons are less branched with fewer release sites, they have lower mitochondrial oxidant stress, and they buffer calcium more robustly.^20^

However, this kind of control mechanism generates superoxide. Superoxide and derivative reactive oxygen species can damage protein, lipid, and DNA. Over time, this stress could irreversibly compromise mitochondrial function. Indeed, loss of mitochondrial complex I function and mitochondrial DNA deletions are hallmarks of the SNc of PD patients.^25^ Mitochondrial oxidant stress in human (but not mouse) DA neurons also increases oxidation of DA, which compromises lysosomal function.^26^ Many PD‐prone neurons have a similar physiological phenotype, including basal mitochondrial oxidant stress. So, the combination of three factors—telencephalization (unequal brain growth), a particular anatomical phenotype, and a physiological phenotype that stresses mitochondria and aging—creates the conditions necessary for genetic mutations and environmental toxins to cause PD specifically in humans.

## Reconciling the Clinical Picture With Theories of Pathogenesis

**Figure 2:**
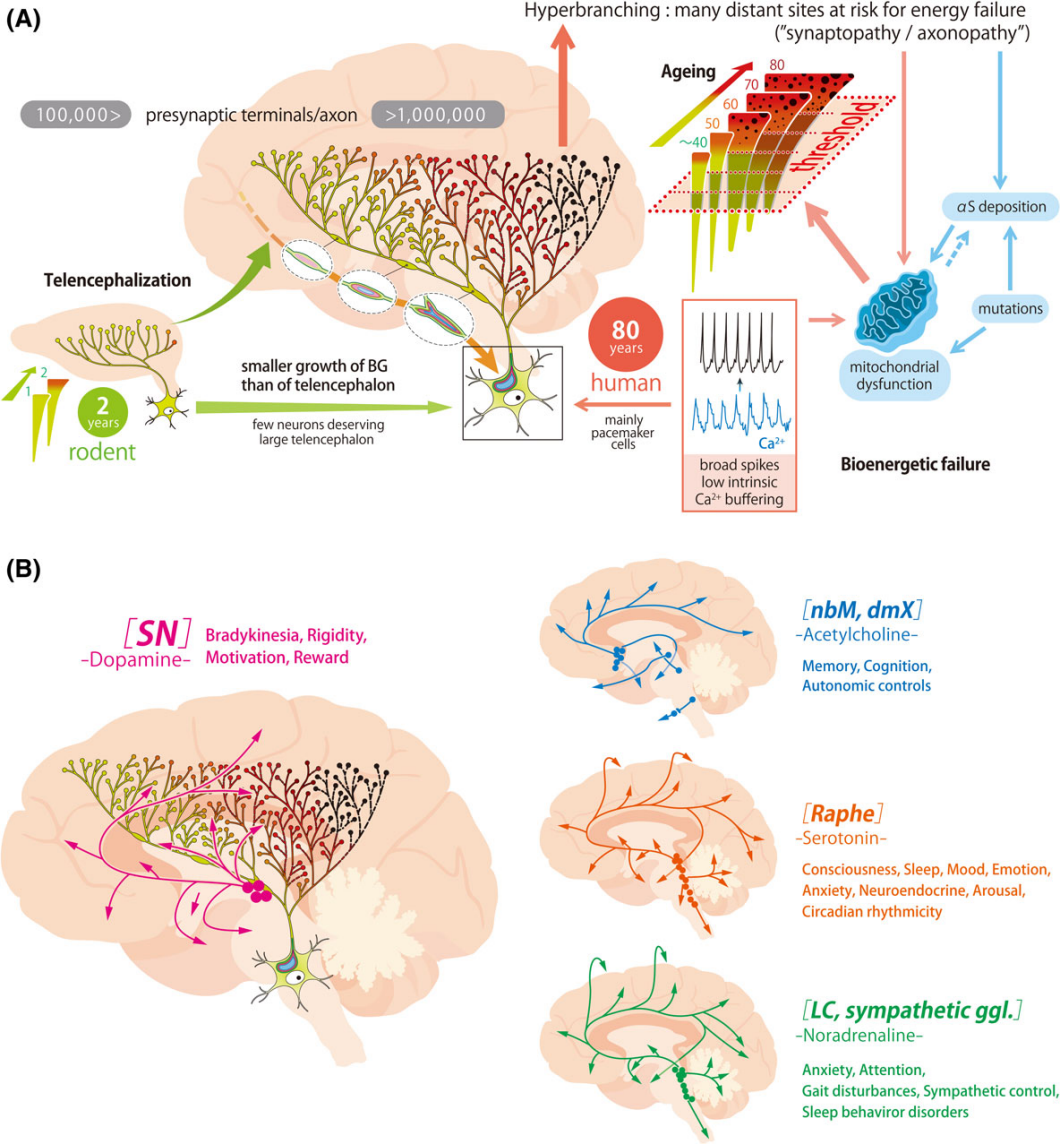
Schematic depicting the key features of system proneness to PD pathology. (A) Telencephalization is exponentially larger in humans than in other mammals, here exemplified as rodents. In parallel, the branching of the BG neurons is considerably larger. Slow pacemaking cells are presumably hyperbranching. Their autonomous cell activity provides long‐range neuromodulation and relies on feed‐forward bioenergetics control, with calcium‐dependent and energy‐expensive cellular metabolism, endangering mitochondria, especially at the α‐synuclein–rich synapses. Once a certain threshold is reached, here schematized as a horizontal bar, synaptic and axonal dysfunction occurs, giving rise to first vague and later more concise clinical symptoms. This cascade of events may occur synchronously in different systems with a similar cellular at‐risk phenotype. The slower growth of the BG in comparison to the telencephalon as well as the long human life span promote these processes. (B) PD‐prone systems are characterized by hyperbranching axons regardless of the neurotranmitters.Because hyperbranching neurons project to wide brain areas, related symptoms are multifaceted. Each dot represents neuronal groups with projecting axons indicated by arrows. Of note, nonmotor symptoms transmitted by the dopaminergic VTA are also included, although disease susceptibility is much higher in SNc than VTA neurons, reflecting the distinctive physiology, bioenergetic control mechanisms, and higher numbers of varicosities in the SNc. nbM, nucleus basalis of Meynert; dmX, dorsal motor nucleus of vagus; ggl., ganglia.

The proposition that PD is a consequence of telencephalization and the pathological stress it places on vulnerable subcortical neuromodulatory networks has clear implications for how symptoms should evolve in PD patients. Our model stands in sharp contrast to the widely held view that PD is a prion disorder.^27^, ^28^ In its simplest form, the prion hypothesis posits that aSYN pathology and symptoms are dictated by synaptic proximity to a peripheral seeding site (in the gut or olfactory bulb); thus, there is a centripetal spread of aSYN pathology through anatomically coupled networks.^29^ This model implies that symptoms should follow a stereotyped sequence, with the earliest symptoms being peripheral. In contrast, the “telencephalization hypothesis” predicts symptoms should manifest themselves in parallel,^30^ with local or patient‐specific factors governing the precise sequence of symptoms (Fig. 2A,B). In this context, our hypothesis is closer to the cortical pathogenic theory of PD proposing that corticostriatal activities figure as “stressors” in parallel to the SNc and other vulnerable structures.^31^

The prion hypothesis is also difficult to reconcile with the variable spatiotemporal distribution pattern of pathology and symptoms observed in PD patients.^32^ This variability in pattern of pathology also prohibits the clear delineation of the sequence and range of PD symptoms (motor and nonmotor). Apathy, fatigue, alexithymia, bradyphrenia, and depression have all been linked to prodromal PD^33^ and are likely to be dependent upon dysfunction in evolutionary old structures coupled to the telencephalon.^32^ At the motor stage of the disease, dysfunction of other evolutionarily old circuitries becomes evident. An example is blindsight, a subconscious form of visual perception considered to be means of detecting rapidly approaching predators. It is impaired in PD patients, because they show deficits in grasping saccades, motion perception, and immediate emotional face perception.^34^ The superior colliculi, which are critical to blindsight, are evolutionarily old structures that are richly connected to the forebrain. PD patients also have deficits in gait automatization. The underlying circuitry in the medulla and spinal cord is evolutionarily old and controlled by telencephalic networks.^35^ Finally, dysregulation of neuromotor systems in sleep may trigger REM sleep behavior disorder, given that in phylogenetic evolution body movements during sleep (active sleep) are primarily a natural phenomenon.^36^

Not all nominally prodromal PD symptoms fall so nicely into our model. Both olfactory and gastrointestinal (GI) dysfunction have been posited to be symptoms of impending PD. Both are thought to be peripheral sites for aSYN pathology. Although these are evolutionarily old systems, the nature of the pathology in these regions, its relationship to nonmotor symptoms, and its relationship to PD more broadly are unresolved. For example, deficits in olfactory discrimination could be a consequence of dysfunction in olfactory cortex, not of lamina propria involvement in the olfactory bulb.^37^ Similarly, the assumption that pathology spreads from the gut to the brain is unproven; it could be that pathophysiology in medullary autonomic control centers are driving GI pathology, or at least that there is a “vicious circle of injury.”^38^

## Future Directions

Our thesis is that PD is a uniquely human disease because of telencephalization and consequent bioenergetic and proteostatic burdens on subcortical structures^37^ that have been “left behind” by human evolution. This stress makes these subcortical structures particularly vulnerable to aging. Our model provides a compelling account of the range and synchronicity of motor and nonmotor symptoms in PD. It provides a ready explanation for the correlation between aSYN pathology and PD. Whereas it does not exclude the possibility that aSYN pathology propagates through synaptically coupled networks in the brain, it argues that this is not a primary driver of pathogenesis in PD. Last, the “telencephalization hypothesis” suggests that disease‐modifying therapies for PD are most likely to come from a better understanding of the unique features of the human brain that drive pathogenesis, like axonal biology, synaptic function in neuromodulatory networks, and neuronal bioenergetics. It is tempting to speculate that there is also evolutionary grounding of other, uniquely human, neurodegenerative processes. Thus, Alzheimer's disease has been seen as the “downside” of the evolution of the human parietal lobe, including the precuneus.^9^ By antagonistic pleiotropy, genetic, molecular, and cellular mechanisms may be contributive as well.^39^

## Author Roles

(1) Research Project: A. Conception, B. Organization, C. Execution; (2) Manuscript Preparation: A. Writing of the First Draft, B. Review and Critique, C. Design of Figures, D. Provision of Further References, E. Final Editing.

N.J.D.: 1A, 1B, 1C, 2A, 2B, 2E

D.J.S.: 1A, 1B, 1C, 2B, 2E

T.U.: 1C, 2B, 2C

S.G.: 1C, 2B, 2D

C.G.G.: 1B, 1C, 2B, 2E

## Financial Disclosures

N.J.D. has received research support by Fondation Think, Rotary Luxembourg, and Rotary International and personal or traveling fees from Alformec (Luxembourg), Bio Expert Nieder‐Olm, and Klinikum Koblenz (both Germany). D.J.S. has received research support from NIH, JPB Foundation, CHDI, MJFF, Flanagan Foundation, and Adamas Pharmaceuticals and personal fees for consulting or advisory board membership from Pfizer, Adamas Pharmaceuticals, and MDS. T.U. has received research support by JSPS KAKENHI (Grant Numbers: 17H03555 and 16 K14572). S.G. has received research support by the Swedish Research Council‐MH‐3026 and HBP‐Horizon 2020. C.G.G. has received research support by NIH, Michael J. Fox Foundation, Parkinson's Disease Foundation, and personal fees from the University of Pittsburgh.
